# Comparison of risk-scoring systems in the prediction of outcome after liver resection

**DOI:** 10.1186/s13741-017-0073-4

**Published:** 2017-11-25

**Authors:** S. Ulyett, G. Shahtahmassebi, S. Aroori, M. J. Bowles, C. D. Briggs, M. G. Wiggans, G. Minto, D. A. Stell

**Affiliations:** 1 Derriford Hospital, Plymouth, PL6 8DH UK; 2 Peninsula Schools of Medicine and Dentistry, Plymouth University, Plymouth, PL6 8BU UK; 3 Nottingham Trent University, Nottingham, NG1 4BU UK

**Keywords:** Preoperative assessment, Liver resection, Surgical complications, Cardiopulmonary exercise testing

## Abstract

**Background:**

Risk prediction techniques commonly used in liver surgery include the American Society of Anesthesiologists (ASA) grading, Charlson Comorbidity Index (CCI) and cardiopulmonary exercise tests (CPET). This study compares the utility of these techniques along with the number of segments resected as predictive tools in liver surgery.

**Methods:**

A review of a unit database of patients undergoing liver resection between February 2008 and January 2015 was undertaken. Patient demographics, ASA, CCI and CPET variables were recorded along with resection size. Clavien-Dindo grade III–V complications were used as a composite outcome in analyses. Association between predictive variables and outcome was assessed by univariate and multivariate techniques.

**Results:**

One hundred and seventy-two resections in 168 patients were identified. Grade III–V complications occurred after 42 (24.4%) liver resections. In univariate analysis of CPET variables, ventilatory equivalents for CO_2_ (VEqCO_2_) was associated with outcome. CCI score, but not ASA grade, was also associated with outcome. In multivariate analysis, the odds ratio of developing grade III–V complications for incremental increases in VEqCO_2_, CCI and number of liver segments resected were 1.09, 1.49 and 2.94, respectively.

**Conclusions:**

Of the techniques evaluated, resection size provides the simplest and most discriminating predictor of significant complications following liver surgery.

## Background

Despite the technical advances, liver resection remains potentially dangerous and is associated with a morbidity rate of 18.2–32.4% (Ulyett et al. 2015; Wiggans et al. 2014; Poon et al. 2004) and mortality rate of 1.4–5.3% (Nygard et al. 2012; Belghiti et al. 2000; Dimick et al. 2004). Preoperative estimation of risk allows counselling of patients regarding treatment options and helps in operative planning. A number of techniques are commonly used preoperatively to estimate risk including the American Society of Anesthesiology (ASA) grade, Charlson Comorbidity Index (CCI) and Cardiopulmonary Exercise Tests (CPET). The ASA grading system is a subjective assessment of the degree of systemic disease made at the time of surgery (Saklad 1941). CCI is a 22-point scoring matrix based on comorbid diagnoses (Charlson et al. 1987), which was originally designed to predict long-term survival in an unselected population but has also been shown to be of value in predicting outcome after surgery (Backemar et al. 2015; Schmolders et al. 2015). CPET provides an objective measurement of cardiorespiratory fitness, where the volume of oxygen consumption at peak (VO_2 peak_) and at anaerobic threshold (AT), ventilatory efficiency in the elimination of carbon dioxide (CO_2_) (VEeqCO_2_), heart rate (HR) and oxygen (O_2_) pulse (a surrogate measure of cardiac output) are measured. This technique was initially used to predict mortality in patients undergoing a range of abdominal procedures (Older et al. 1993) and has been shown to be of value in predicting outcomes in patients undergoing pancreatic (Chandrabalan et al. 2013) and vascular (Thompson et al. 2011) surgery.

Data on the use of these tools in the context of liver surgery is scarce. The ASA grade has been shown to influence the development of postoperative complications after liver resection (Belghiti et al. 2000), and CCI has been assessed in the prediction of short-term outcomes (Schroeder et al. 2006). Data on the use of CPET before liver surgery is conflicting, with one study showing a useful correlation with complications (Junejo et al. 2012) and another showing only minimal association (Dunne et al. 2014). None of the tools takes into account the extent of the proposed operation, and no comparison between the techniques has been undertaken.

The aim of this study is to determine the relative value of these risk prediction tools in patients undergoing liver surgery and also to assess their value compared to risk prediction based on the extent of the surgical procedure undertaken.

## Methods

A review of a prospectively maintained database of all patients undergoing resection of parenchymal liver lesions between February 2008 and January 2015 was undertaken. Follow up was completed in June 2015. The primary endpoint was development of Clavien-Dindo (CD) grade III–V complications.

To reduce heterogeneity of the study population, patients undergoing synchronous bowel resection or surgery for obstructing lesions of the proximal hepatic duct or in the presence of liver cirrhosis were excluded, as surgery in these situations is associated with higher risk (Wiggans et al. 2014; Belghiti et al. 2000; Das et al. 2001). Liver resection was undertaken using Cavitron Ultrasonic Surgical Aspirator (CUSA). General anaesthetic was administered by specialist liver anaesthetists. Low central venous pressures (CVP) were maintained although invasive CVP monitoring was not undertaken. The extent of liver resection was described according to the Brisbane Classification (Pang 2002). Radiofrequency ablation (RFA) was used where major liver resections were performed leaving a residual contralateral disease. Where sub-segmental resections were undertaken, these were rounded up to the nearest integer in analyses. Postoperatively, all patients undergoing major resection were cared for in a critical care unit. Retrieved data include age, gender, indication for surgery and use of preoperative chemotherapy. ASA grade was determined at the time of surgery by the responsible anaesthetist, and CCI was calculated postoperatively from clinical records. The use of perioperative blood transfusion was also recorded.

CPET was introduced as a preoperative assessment tool in February 2008 and was initially available at the discretion of referring consultants for patients considered to be at higher risk of surgical complications. After November 2013, CPET has been undertaken in the majority of patients. CPET was undertaken using a cycle ergometer (nSpire™ Zan® 600, Colorado, USA, or MGC Diagnostics®, MN, USA). The protocol consists of 2 mins of rest, 1 min of cycling without resistance, and then a ramped protocol of between 10 and 25 watts/min. CPET variables measured included VO_2 peak_, anaerobic threshold (AT), O_2_ pulse, relative O_2_ pulse, resting (rHR) and peak heart rate (pHR) and ventilatory equivalents for CO_2_ (VEqCO_2_). AT was calculated using the V-slope method and VO_2 peak_ was averaged over the last 30 s of the test. CPET were undertaken and interpreted by three specialist liver anaesthetists. In patients where AT was not achieved, a nominal low value of 8 ml/kg/min was assigned as this group have been shown to have poor outcomes (Lai et al. 2013; Challand et al. 2012).

Postsurgical outcomes occurring within 30 days of surgery were classified according to the CD system (Dindo et al. 2004). Broadly, grade I–II complications include minor variations in the patient pathway including the use of anti-emetics and antibiotics, grade III complications require postoperative intervention (commonly for bile leaks), grade IV complications are determined by organ failure and grade V complication is death. In this study, grade III–V complications were used as a composite outcome of significant adverse postoperative events. Patients may develop complications in more than one grade, particularly as grade III and IV complications may have different aetiology. Liver failure was classified according to the International Study Group for Liver Surgery consensus definition of post-hepatectomy liver failure (PHLF) (Rahbari et al. 2011) and renal failure according to the RIFLE scoring system (Bellomo et al. 2004). For the purposes of this study, heart failure was defined as the requirement for inotropic medication to treat hypotension of suspected cardiac cause, after removal of epidural catheter. Respiratory failure was defined as a return to critical care unit for respiratory support.

Statistical analyses were carried out using chi-square and Mann-Whitney *U* tests for categorical and continuous data, respectively. Binary logistic regression was used to assess the effect of risk factors on outcome. Repeat resections in individual patients were analysed separately where separate CPET were performed.

Confirmation was obtained from the South-West Health Research Authority that Research Ethics Committee review was not required because patient data were collected prospectively as a normal part of hospital care, and all data were anonymised. No patient consent was required for this study. This study was registered with the Research Registry (unique identification 464) (Research Registry) and conforms with the STROBE guidelines (von Elm et al. 2008).

## Results

**Fig. 1:**
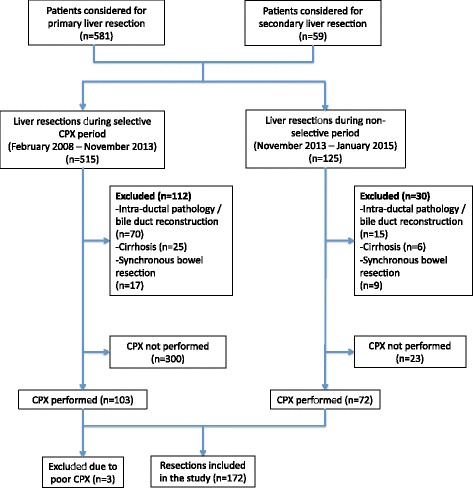
Study population derived from 640 patients considered for liver resection between February 2008 and January 2015

Details of patients selected for the study are shown in Fig. 1. Nine (5%) patients had repeat resections. Patient and operative characteristics, CPET parameters and ASA and CCI scores are shown in Table 1. VEqCO_2_ results were unavailable in five patients. Intraoperative RFA was used in addition to resection in 11 patients and portal vein embolization prior to resection in three patients. Laparoscopic resection was performed on 32 (18.6%) occasions.

**Table 1:** Baseline patient characteristics

Age (median [range])	69 (22–90)	CPET parameters	
Gender (%)			
Male	119 (69.2)	VO_2_ at AT (ml/kg/min, [range])	12.5 (5.6–23.1)
Female	53 (30.8)		
Diagnosis (%)			
Colorectal metastases	134 (77.9)	VO_2 peak_ (ml/kg/min, [range])	18.9 (6.4–35.5)
Hepatoma	12 (7)		11.8 (5–25.3)
Neuroendocrine tumour	6 (3.5)	Oxygen pulse (ml/beat, [range])	
Other	20 (11.6)		
ASA (%)			
I	8 (4.7)		14.9 (7.8–26.6)
II	93 (54.1)	Relative oxygen pulse (100 ml/beat/kg, [range])	
III	70 (40.7)		
IV	1 (0.6)		
CCI (median [range])	4 (0–9)	Resting heart rate (beats/min, [range])	77 (39–119)
Preoperative chemotherapy (%)		Peak heart rate (beats/min, [range])	132 (79–180)
Yes	62 (36)		29.6 (17.1–49.9)
No	94 (54.7)		
Unknown	16 (9.3)	VEeqCO_2_ at AT (range)	
RFA used (%)			
Yes	11 (6.4)		
No	161 (93.6)		
Number of segments resected (%)			
1	46 (26.7)		
2	13 (7.6)		
3	14 (8.1)		
4	64 (37.2)		
5	31 (18)		
6	4 (2.3)		

Demographic and operative characteristics of 172 liver resections in 168 patients who had preoperative CPET

*RFA* radiofrequency ablation

A surgeon estimated that the intraoperative blood loss was less than 500 ml in 81 (47%) and more than 1000 ml in 31 (18%) resections. Forty-four patients required a blood transfusion intraoperatively with a median transfusion of 2 units (1–18) for those transfused. Clavien-Dindo grade III to V complications occurred following 42 (24%) of 172 resections (Table 2). Eleven patients suffered both III and IV/V complications. One patient developed heart failure secondary to cardiac arrhythmia requiring cardiac pacing. There were no cases of postoperative respiratory failure. The proportion of patients suffering grade III–V complications in the selective and non-selective CPET periods was similar (22 and 27%, respectively).

**Table 2:** Summary of complications after liver resection

Grade III complications (%)	15 (8.6)
• Bile leak requiring ERCP	3 (1.7)
• Bile leak requiring drain	4 (2.3)
• Infected collection requiring drain	1 (0.6)
• Further surgery required	2 (1.1)
• Open drainage of collection following colonic injury,	
• Laparotomy undertaken for ileus	
• Pneumothorax requiring drain	1 (0.6)
• Cardiac pacing	1 (0.6)
• Ascites requiring drain	1 (0.6)
• Pleural effusion requiring drain	1 (0.6)
• Gastric burn secondary to radiofrequency ablation	1 (0.6)
Grade IV and V complications (%)	37 (21.3)
• Liver failure alone	24 (13.8)
• Renal failure alone	6 (3.4)
• Liver and renal failure	6 (3.4)
• Heart failure	2 (1.1)
• Death	2 (1.1)

Summary of Clavien-Dindo grade III–V postoperative complications following 172 liver resections in 168 patients. Some patients suffered more than one complication

**Fig. 2:**
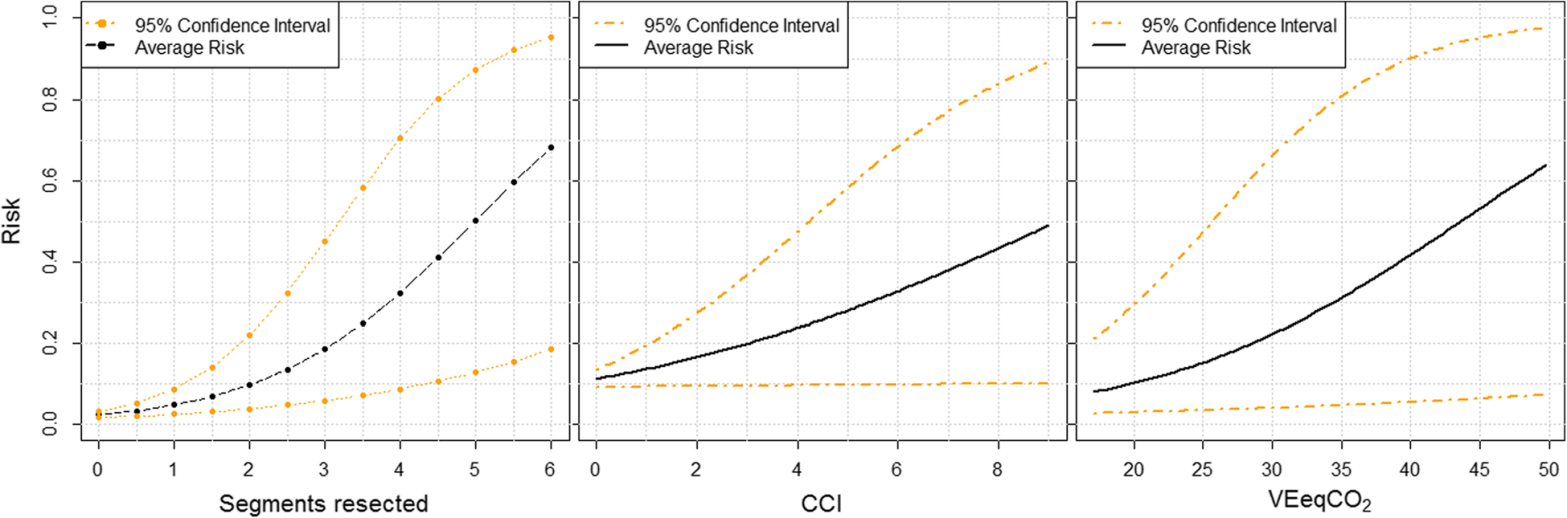
Association of **a** extent of liver resection, **b** CCI score and **c** VEqCO_2_ with the risk of developing Clavien-Dindo grade III–V complications following 172 liver resections. N.B. The number of liver segments resected was analysed as a discrete variable, whereas the CCI and VEqCO_2_ were analysed as continuous variables

Demographic details, ASA, CCI, use of pre-operative chemotherapy, CPET variables and the number of liver segments resected in patient groups with and without significant postoperative complications are shown in Table 3. In univariate analysis CCI score, but not ASA grade, was associated with grade III–V complications (*P* = 0.02). Of the CPET variables, VEeqCO_2_ was associated with the development of grade III–V complications (*P* = 0.005). AT was not detectable in three patients due to very limited exercise tolerance, and none of whom suffered grade III–V complications. The number of liver segments resected was strongly associated with outcome (Fig. 2). The use of preoperative chemotherapy was not associated with postoperative complications.

**Table 3:** Comparison of potential risk factors for developing complications

Variable (median)	CD 0–II (*n* = 130)	CD III–V (*n* = 42)	*P* value
Age (range)	69 (22–90)	70 (49–88)	0.47
Gender (%)			0.057
Male	85 (65.4)	34 (81)	
Female	45 (34.6)	8 (19)	
ASA (%)			0.187
I–II	80 (61.5)	21 (50)	
III–IV	50 (38.5)	21 (50)	
CCI (range)	4 (0–7)	5 (1–9)	*0.021*
Preoperative chemotherapy (%)			0.85
Yes	46 (35.4)	16 (38.1)	
No	71 (54.6)	23 (54.8)	
Unknown	13 (10)	3 (7.1)	
Number of segments resected (range)	3 (1–6)	4 (3–6)	*< 0.001*
VO_2_ at AT (ml/kg/min, [range])	12.8 (6.4–22.9)	12.5 (5.6–23.1)	0.84
VO_2 peak_ (ml/kg/min, [range])	18.8 (6.4–35.5)	19.2 (11.8–30.8)	0.65
Oxygen pulse (ml/beat, [range])	11 (5–20.5)	12.3 (5–25.3)	0.39
Relative oxygen pulse (100 ml/beat/kg, [range])	15 (7.8–26.6)	14.9 (10–24.5)	0.52
Resting heart rate (beats/min, [range])	74 (39–115)	80 (43–119)	0.54
Peak heart rate (beats/min, [range])	131.5 (79–180)	133.5 (85–172)	0.89
VEeqCO_2_ at AT (range)	29.1 (17.1–49.9)	31.7 (24.4–46.2)	*0.005*

Univariate analysis of association of age, gender, diagnosis, ASA grade, CCI, preoperative chemotherapy, extent of resection and CPET values with Clavien-Dindo 0–II and III–V complications following 172 liver resections

Multivariate analysis of predictive scores and the extent of liver resection shows that the extent of resection, VEqCO_2_ and CCI are independently associated with the development of grade III–V complications (Table 4). ASA grade was not associated with outcome. The strongest association with complications was shown to be with the extent of liver resection, where each extra segment of liver resected increased the odds of developing CD grade III–V complications by a factor of 2.94 (1.86–4.66). The greatest range of predictive values (17–50) was noted for VEqCO_2,_ where an incremental increase of 1.09 was noted in the OR of developing grade III–V complications.

**Table 4:** Comparison of risk assessment techniques

	OR	95% CI	*P* value	Range of predictive values
ASA grade	0.85	0.37–1.94	0.7	1–4
CCI score	1.49	1.09–2.04	0.01	0–9
VEeqCO_2_ at AT	1.09	1.01–1.17	0.04	17–50
Number of liver segments resected	2.94	1.86–4.66	< 0.001	1–6

Multivariate analysis of risk assessment techniques in the prediction of CD grade III–V complications compared with grade 0–II complications following 172 liver resections

## Discussion

The main finding of this study is the very significant association between postoperative complications in liver surgery and the extent of the liver resection undertaken. There is a weaker association between postoperative complications and risk scores formulated by assessment of recorded comorbidity (CCI) and CPET parameters. Subjective assessment of a patient’s fitness at the time of surgery by ASA score is not predictive of postoperative complications.

The scoring systems under assessment in this study were chosen because they assess risk by different techniques. CPET is an objective measure of cardiovascular fitness, CCI scores are derived from recorded comorbidity, ASA grade is a subjective assessment of overall health and the number of liver segments resected provides a simple measure of operative extent. Assessment of the techniques in parallel allows a comparison of their relative utility.

The role of CPET in liver surgery is yet to be established, with conflicting outcomes from the two published studies investigating the technique (Junejo et al. 2012; Dunne et al. 2014). CPET is useful in surgery where cardiorespiratory complications form a major part of adverse outcomes, such as vascular (Thompson et al. 2011; Elkouri et al. 2004) and cardiothoracic (Van Diepen et al. 2014) surgery. As the main cause of morbidity and death after liver surgery is liver failure (Poon et al. 2004; Belghiti et al. 2000; Jarnagin et al. 2002), the degree of cardiovascular fitness is likely to have a weaker association with outcome. In our series, significant cardiovascular complications were rare, occurring in only two patients. Also, patients undergoing liver resection may be a selected group with better physical function, as many will have previously undergone, and recovered from, primary colorectal surgery. AT was originally shown to be useful in predicting mortality in a large, unselected population undergoing a range of elective abdominal procedures including vascular surgery, in which 24% of patients had evidence of pre-existing cardiovascular disease (Older et al. 1993), and mortality in this context is more likely to be related to cardiovascular health.

Of the CPET parameters under study, VEqCO_2_ was shown to be predictive of postoperative complications, in keeping with earlier findings (Junejo et al. 2012). Although the incremental OR for predicting CD grade III–V complications is low (1.09), this effect is noted over a large range of values (17–50). Despite other studies demonstrating the value of AT in the prediction of outcomes after liver resection (Dunne et al. 2014), this parameter was not shown to be of value in this series, although many of the CPET variables are mathematically related, and the difference in predictive value between them may be less than is apparent in a multivariate analysis. In a similar manner to CCI, the median VEqCO_2_ was very similar between groups with CD grade 0–II and III–V complications (29.1 vs 31.7) with significant overlap in range, and this may limit the usefulness of this test. ASA was not shown to predict outcome in multivariate analysis compared with the other measures. ASA is known to be a highly subjective tool, with significant inter-observer variation (Mak et al. 2002; Ranta et al. 1997). In practice, this is also too blunt a tool to be of value, as 95% of patients have ASA score of II or III.

The CCI is a well-researched measure used to weight outcomes in cancer surgery (Dobbins et al. 2015). The tool has also been used in registry data when comparing outcomes between individual hospitals (Dobbins et al. 2015) and clinicians (Ugolini and Nobilio 2004). Its role in predicting specific surgical complications is variable. It has not been shown to be associated with complications in gynaecologic or colorectal surgery (Suidan et al. 2015; Krarup et al. 2015), but is associated with outcome in orthopaedic surgery (Schmolders et al. 2015). While CCI is associated with complications in this study, it is less predictive of outcome over the range of measured values than either the number of liver segments resected or VEqCO_2_ (Fig. 2). Although CCI provides a simple measure that can be easily calculated with knowledge of a patient’s medical history, the median score of patients suffering grade III–V complications is only one point higher than those with grade 0–II complications, with a large overlap in the score range which limits the usefulness of the technique. Also, the CCI system records comorbidity rarely relevant in the context of elective liver surgery, including AIDS and lymphoma, while other more common potential risk factors, such as extremes of BMI (Vigano et al. 2011; Balzan et al. 2010) and NAFLD (Wakai et al. 2011), are not included.

The factor with the greatest predictive value for outcome in this analysis is the extent of the liver resection undertaken, which may be expected as liver failure due to insufficient liver volume is the major cause of death after liver surgery (Wiggans et al. 2013). The high odds ratio of 2.94 in the prediction of grade III–V complications for increasing number of resected segments make this a factor of high clinical relevance and easily utilised, as the extent of liver resection to be undertaken is usually known preoperatively. Of note, the increased risk of liver resection with increasing number of resected segments is non-linear, with the largest absolute increase in risk being experienced by patients undergoing resection of five and six liver segments.

Each of the three techniques shown to be of value has very wide confidence intervals in the prediction of outcome (Fig. 2). The potential risk faced by patients undergoing liver surgery is affected by all of the factors under study (comorbidity, physical functioning and extent of liver resection), and each of the tests used individually therefore will be limited in their predictive value. Other factors are also likely to influence operative risk, particularly the presence of a coexisting liver disease. A useful area of further study would be to develop a compound risk scoring system based on all these factors.

The study may be subject to bias due to patient selection. Throughout the series, CPET has been undertaken on patients perceived to be less healthy, which accounts for the male predominance of the population under study. It is unlikely however that CPET parameters would be more predictive of complications in a healthier population. A potential limitation of this study is the degree of confounding caused by prior awareness of CPET results by clinicians, as it is possible that patients with low levels of fitness were treated differently. Preoperative medication which may affect CPET results has not been recorded. However, in undertaking CPET, it is important to estimate functional parameters as they would be at the time of surgery, including the influence of medication.

## Conclusions

This study represents a large case-series, which attempts to answer the question of how best to assess risk in patients undergoing liver resection. The simplest factor to consider is the extent of the planned liver resection. CCI and CPET parameters may be useful discriminators for potential risk in patients undergoing the same resection type and may also contribute to decision-making in patients who require extended liver resections.

## Abbreviations

AIDS: Acquired immunodeficiency syndrome
ASA: American Society of Anesthesiology
AT: Anaerobic threshold
BMI: Body mass index
CCI: Charlson Comorbidity Index
CD: Clavien-Dindo
CPET: Cardiopulmonary Exercise Testing
HR: Heart rate
NAFLD: Non-alcoholic fatty liver disease
O_2_: Oxygen
PHLF: Post-hepatectomy liver failure
RFA: Radiofrequency ablation
VEeqCO_2_: Ventilatory efficiency in the elimination of carbon dioxide
VO_2 peak_: Volume of oxygen consumption at peak
